# FastDMA: An Infinium HumanMethylation450 Beadchip Analyzer

**DOI:** 10.1371/journal.pone.0074275

**Published:** 2013-09-05

**Authors:** Dingming Wu, Jin Gu, Michael Q. Zhang

**Affiliations:** 1 Bioinformatics Division/Center for Synthetic and Systems Biology, Tsinghua National Laboratory for Information Science and Technology (TNLIST), Department of Automation, Tsinghua University, Beijing, China; 2 Department of Molecular and Cell Biology, Center for Systems Biology, The University of Texas at Dallas, Dallas, Texas, United States of America; University of Southern California, United States of America

## Abstract

DNA methylation is vital for many essential biological processes and human diseases. Illumina Infinium HumanMethylation450 Beadchip is a recently developed platform studying genome-wide DNA methylation state on more than 480,000 CpG sites and a few CHG sites with high data quality. To analyze the data of this promising platform, we developed FastDMA which can be used to identify significantly differentially methylated probes. Besides single probe analysis, FastDMA can also do region-based analysis for identifying the differentially methylated region (DMRs). A uniformed statistical model, analysis of covariance (ANCOVA), is used to achieve all the analyses in FastDMA. We apply FastDMA on three large-scale DNA methylation datasets from The Cancer Genome Atlas (TCGA) and find many differentially methylated genomic sites in different types of cancer. On the testing datasets, FastDMA shows much higher computational efficiency than current tools. FastDMA can benefit the data analyses of large-scale DNA methylation studies with an integrative pipeline and a high computational efficiency. The software is freely available via http://bioinfo.au.tsinghua.edu.cn/software/fastdma/.

## Introduction

DNA methylation is an important type of epigenetic modification in the human genome. The function of DNA methylation is traditionally described as silencing mark [1]. Recent studies suggest DNA methylation plays more sophisticated roles in chromatin structure and transcriptional regulation [2]. DNA methylation is highly dynamic but under strict control during development [3], [4]. While in human diseases, especially in cancer, the DNA methylation states are usually significantly disrupted and those changes are strongly associated with cancer hallmarks [5]–[7].

There are four popular techniques for detecting genome-wide DNA methylation state: whole genome bisulphite sequencing [8], methylation array [9], reduced representation bisulfite sequencing [10] and enrichment based method [11]. Infinium HumanMethylation450 Beadchip is a recently developed methylation array platform which detects more than 480,000 cytosine sites along the entire human genome. It covers the majority of reference genes and shows high data reproducibility between technical replicates. Because of the lower cost and easier experimental protocol, this platform is suggested to be suitable for large-scale studies [12]. The Cancer Genome Atlas (TCGA) program, which aims to providing a comprehensive molecular portraits of all types of cancer, has already used this platform to profile the DNA methylation states of hundreds of clinical samples [13], [14].

Here we propose our recently developed software, FastDMA, to help researchers to analyze the data generated by this platform, especially for large or clinical datasets. FastDMA uses a unified statistical model, analysis of covariance (ANCOVA), to do both single probe analysis and differentially methylated region (DMR) scanning. Technically, FastDMA is implemented as a standalone software in C++ which can be easily distributed and further developed. Besides, by using parallel computing technique, it can deal with large-scale datasets with very high computational efficiency.

This article is organized as follows: in the Methods section, we described the workflow, the computational model and the software implementation of FastDMA. In the Results section, we first applied FastDMA on three large-scale datasets from TCGA of breast invasive carcinoma (BRCA), lung adenocarcinoma (LUAD), and prostate adenocarcinoma (PRAD). And then, we compared FastDMA with a recently published software IMA [15] for both the correctness and the computational performance. Finally, we discussed the major advantages of the software and its limitations waiting for further developments.

## Methods

### Overview

**Figure 1:**
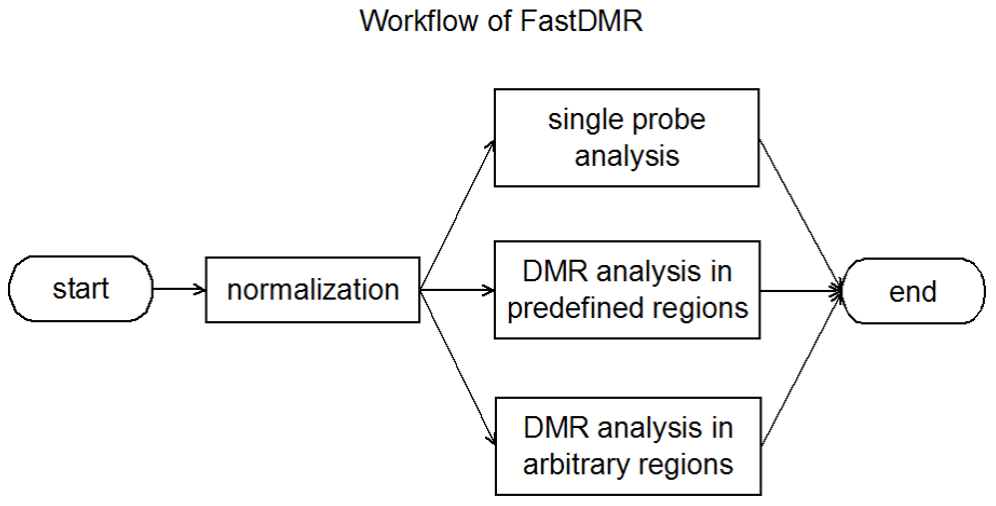
Workflow of FastDMA.

The workflow of FastDMA is shown in Figure 1. FastDMA can do data normalization and support both single probe and region based data analyses. The input of FastDMA is usually a data matrix, processed from the original fluorescence signal, containing the beta value (a value indicating the DNA methylation level, between 0∼1) and the detection pvalue (a value indicating whether the signal is believable or not) of each probe on the beadchip. For the outputs, FastDMA generated human-readable table-limited text files and the formatted BED files compatible for UCSC Genome Browser visualization.

Except for the case-control comparison, multiple-group comparison is usually required. For example, it is required to compare the DNA methylation levels in three groups if we want to identify the differentially methylated sites among normal, primary tumor, and metastasis samples. Besides, other clinical co-variables (such as sex, age, etc.) rather than the group labels should also be considered. To deal with these issues in a unified framework, FastDMA used ANCOVA as the main statistical model, a generalized linear model combining ANOVA and regression.

For clearly presenting the statistical model, we denoted some variables as follows. The dataset contains *n* groups, G_i_, (1≤*i*≤*n*), to be compared. For *i*-th group G_i_, there are *m_i_* samples, S_ij_ (1≤*j*≤*m_i_*). The *k*-th probe of sample S_ij_ has a methylation level (as beta-value) β_ijk_. Clinical information (such as sex, age, etc.) is treated as the co-variable: C_ijl_ stands for the value of the *l*-th co-variable of sample S_ij_. Gaussian noise is assumed and denoted as *e*. FastDMA rejects the data points with detection pvalues greater than 0.05 (unreliable data points).

### Normalization

The normalization task assumes that all the studied samples have the same overall beta value distribution. FastDMA uses quantile normalization [16]: first, calculate the quantiles of beta values (logit transformed) in all the samples; then, calculate the averages of the quantiles; and finally, piecewise linear transformation is applied on each beta value such that every sample has the same quantiles as the average. Users can do normalization with any other methods by themselves and skip this step.

### Single Probe Analysis

The first task is to identify the significantly differentially methylated probes. The null hypothesis is that if a probe is not differentially methylated, the beta values of that probe have the same mean values among all the groups. Otherwise, different means among the groups are required. ANCOVA is used in this task to compare two linear regression models that one assumes an overall mean across all the studied groups while the other assumes different means. For the *k*-th probe of sample S_ij_ belonging to group G_i_, we have:

The null hypothesis is formulated as
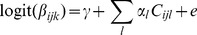
(1)


The alternative hypothesis is formulated as
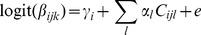
(2)


C_ijl_ stands for the value of the *l*-th co-variable of sample S_ij_. The middle terms in both equations are the effect of the co-variables. It is assumed to be linear.

ANCOVA compares these two linear regression models and gives a pvalue indicating to what extent one can reject the null hypothesis. Then, false discovery rate (FDR) is calculated according to Benjamini–Hochberg (BH) procedure. The pvalue and FDR thresholds to call significantly differentially methylated probes can be manually adjusted by users. To facilitate the following functional analysis, the probes are annotated (such as nearby genes) using a well-curated database IlluminaHumanMethylation450k.db (Tim Triche and Jr. R package version 1.4.6.).

### DMR Analysis in Predefined Regions

Single differentially methylated probe may be hard to be interpreted biologically. FastDMA provides region-based analysis, which can provide additional information. For a predefined region (such as a promoter or a CpG island) containing several probes, if the region is uniformly methylated among all the groups, then it is assumed that the beta values of every probe in that region are distributed with the same means independent with their group labels. Otherwise, the region is called to be differentially methylated. For the *n*-th region, there are *r* probes located in it denoted as P_1_, P_2_,…, P_r_. The beta value of the probe P_k_ of the sample S_ij_ is denoted as 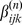 to emphasize that the probe belongs to the *n*-th region.

Then the null hypothesis for this region is formulated as
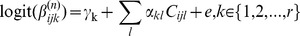
(3)


The alternative hypothesis is formulated as
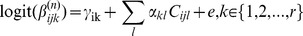
(4)


Similar to the comparison of the two models in the single probe analysis, ANCOVA can compare the above two series of models and calculate the pvalues to evaluate their differences fitting the same data. And also, FDR is calculated using BH procedure.

### DMR Analysis in Arbitrary Regions

In the second task, users should predefine the regions. FastDMA can also scan all the regions covered by probes in the whole genome. The idea to find arbitrary DMRs is to use a sliding window across the genome and test whether each sliding window is a DMR using the above statistical model. Then the overlapped DMRs are merged to give the final results. The default window size is set as 200 bp and only the windows with more than 2 probes are used in the analysis.

It is time-consuming to fit the models in ANCOVA of all the sliding windows across the whole genome. FastDMA provides another intuitive way to deal with this task: it directly uses the FDRs of the probes in a sliding window from the single probe analysis and simply calculates their geometric mean as the score of that region. Windows with this score less than 0.05 are treated as DMRs and the overlapped DMRs are combined as the final results.

### Implementation

The computerized procedures are coded in C++. An additional script for preprocessing the format of the data is coded in Python. The software follows the GNU general public license version 3 for academic use and can be downloaded via the site http://bioinfo.au.tsinghua.edu.cn/software/fastdma. Both a simplified procedure and the advanced user manual to install and run FastDMA are provided on the website.

## Results

### Datasets and Experiments

We applied FastDMA on three large-scale Infinium HumanMethylation450 Beadchip datasets from TCGA of BRCA, LUAD, and PRAD. The numbers of samples for the datasets are 120, 69, and 225 respectively.

### Many Probes are Found Differentially Methylated in Tumor

**Figure 2:**
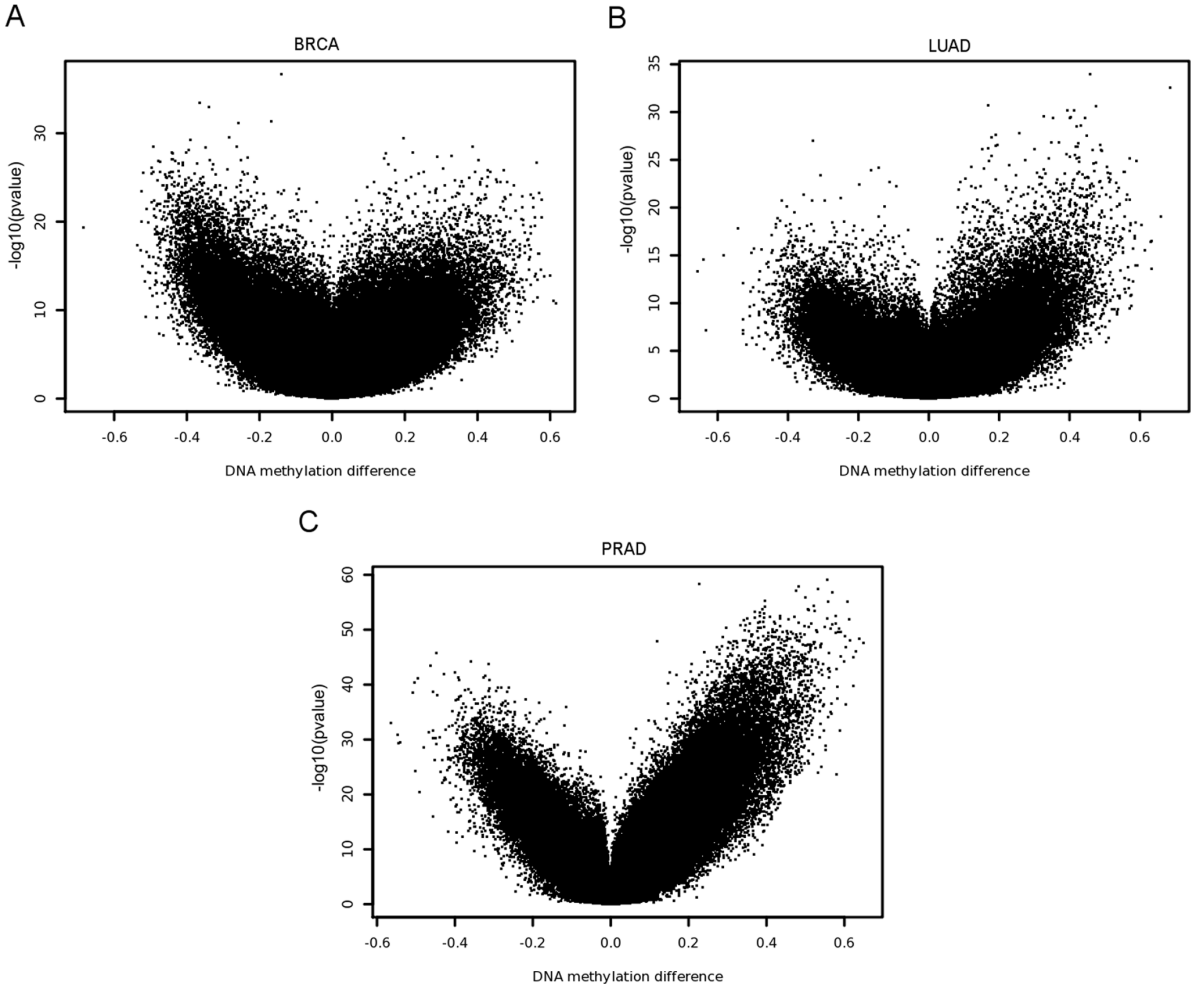
Volcano plots of single probe analysis. Panel A, B, and C shows the result on BRCA, LUAD and PRAD, respectively. Each dot in the plot represents a beadchip probe. The vertical axis indicates the log-transformed pvalue of that probe calculated by ANCOVA and the horizontal axis indicates the mean difference between the methylation levels in tumor and normal samples (tumor - normal).

FastDMA was used to calculate the pvalue and the absolute difference of the differential DNA methylation level of each probe. The results of the overall statistics are shown as volcano plots in figure 2. The top 10 tumor-hypermethylated probes are summarized in table 1. As we can see in figure 2, many probes are significantly differentially methylated. We set the threshold as pvalue<0.01 and FDR<0.05. FastDMA identifies about half of the probes as differentially methylated ones in BRCA and PRAD datasets. Although less than those two, the LUAD dataset still has more than one third of the probes identified as differentially methylated ones (detailed numbers are shown in table 2). These numbers agree with a recently published study on liver hepatocellular carcinoma [17]. These results imply that the cancer genome is globally changed in the consideration of DNA methylation state.

**Table 1:** The top 10 hypermethylated probes in the three cancer datasets.

BRCA			LUAD			PRAD		
ID	pvalue	gene	ID	pvalue	gene	ID	pvalue	gene
cg08370082	3.56E-30	SENP5	cg14001664	1.05E-34	–	cg24922143	7.51E-60	–
cg006921703	3.48E-29	ZYG11B	cg08566455	2.66E-33	–	cg26075747	5.27E-59	HLA-E
cg12669271	1.39E-28	EXT1	cg02627286	2.16E-31	SPG7	cg05063999	1.38E-58	–
cg02953927	1.92E-28	–*	cg22167515	2.61E-31	–	cg00970396	3.26E-58	–
cg15174623	3.50E-28	–	cg12595013	6.04E-31	ZIC1	cg01940855	8.09E-58	CHST11
cg08138586	4.59E-28	PARD3B	cg24722073	6.75E-31	–	cg09296001	1.36E-57	SND1
cg24699719	7.53E-28	–	cg09471659	2.55E-30	–	cg00800229	1.17E-56	SALL2
cg09881545	1.07E-27	–	cg20019985	3.45E-30	–	cg05372113	4.72E-56	CYP2A13
cg23302649	1.95E-27	C14orf23	cg08857994	4.06E-30	–	cg24033558	6.84E-56	SHF
cg19870567	3.38E-27	–	cg14823851	4.34E-30	TBX4	cg00817367	8.66E-56	GRASP

* No gene is annotated related to this probe.

**Table 2:** The numbers of differentially methylated probes identified by FastDMA and IMA.

		Total		Tumor-hypermethylated		Tumor-hypomethylated	
		IMA	FastDMA	IMA	FastDMA	IMA	FastDMA
BRCA	Total	227,221	232,648	110,094	111,579	117,127	121,069
	Overlap	225,292		108,667		116,625	
	Genic	152,445	156,088	80,163	81,266	72,282	74,822
	Island	64,854	66,422	49,998	51,014	14,856	15,408
	Promoter	35,332	36,238	24,904	25,582	10,428	10,656
LUAD	Total	176,982	178,028	80,461	81,209	96,521	96,819
	Overlap	175,693		79,994		95,699	
	Genic	117,158	118,058	57,601	58,206	59,557	59,852
	Island	57,120	57,801	45,659	46,277	11,461	11,524
	Promoter	28,682	29,096	19,818	20,303	8,864	8,793
PRAD	Total	228,366	229,458	120,700	120,638	107,666	108,820
	Overlap	227,478		120,194		107,284	
	Genic	148,248	148,989	81,875	81,871	66,373	67,118
	Island	56,566	56,979	38,214	38,210	18,352	18,769
	Promoter	28,471	28,832	11,588	11,577	16,883	17,255

The thresholds for the two softwares to identify differentially methylated probes are all set as pvalue <0.01 and FDR <0.05. Each cell shows the number of differentially methylated probes identified in that specified condition. Overlap: number of probes found by both FastDMA and IMA; Genic: genic region; Island: CpG island region; Promoter: promoter region. FastDMA and IMA find more than 98% probes the same.

To illustrate the support for multiple-group and clinical co-variable, we constructed a small-scale testing dataset including the array data of four BRCA metastatic tumor samples and randomly chosen size-matched primary tumor samples and normal samples from TCGA. The age at initial pathologic diagnosis is treated as the co-variable. We ran FastDMA to find the differentially methylated sites across normal, tumor and metastatic groups. With the threshold pvalue<0.01 and FDR<0.05, 29 probes are identified as differentially methylated (this small number is mainly due to too few available samples).

### DMR Analysis Helps to Find Genomic Regions for Further Study

**Figure 3:**
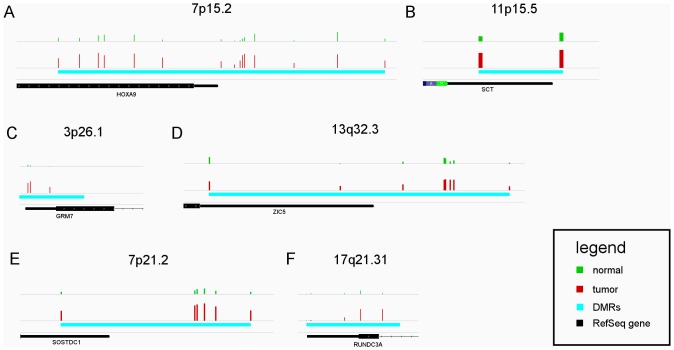
Several examples for the genomic regions identified as DMRs by FastDMA. All genomic regions shown in this figure are hypermethylated in cancer and lie in the 5′ region of a nearby gene. The green (normal) and red (tumor) vertical bars represent the methylation levels of the probes located in each region. The cyan horizontal bars show the differentially methylated regions identified by FastDMA. A, B are from BRCA dataset; C, D are from LUAD dataset; E, F are from PRAD dataset.

The DMR scanning can be done either in predefined regions or in arbitrary regions across the whole genome using the same computational model. Here, we only reported the results by scanning arbitrary regions. The statistical significance thresholds were set the same as the single probe analysis (pvalue <0.01 and FDR <0.05). Under such settings, FastDMA founds 60877, 48930, and 56179 DMR candidates (mainly spanning several hundred base pairs) in the BRCA, LUAD, PRAD datasets respectively. Table 3 shows the state (tumor-hypermethylated or tumor-hypomethylated) and location (CpG island or non-CGI) of these DMR candidates. Figure 3 is a collection of IGV [18] screenshots showing several cases. All these cases are hypermethylated in cancer and lie in the 5′ region of a nearby gene (promoter region). These regions contain dense distribution of many differentially methylated probes and therefore can be interesting candidates for further functional or biomarker analysis.

**Table 3:** Summarization of the DMR candidates identified by FastDMA.

		CpG island	non-CGI
BRCA	tumor-hypermethylated	23872	9771
	tumor-hypomethylated	6362	20871
LUAD	tumor-hypermethylated	20525	6404
	tumor-hypomethylated	5320	16680
PRAD	tumor-hypermethylated	16471	12631
	tumor-hypomethylated	9421	17655

To validate our results, we randomly selected 20 candidates in the LUAD datasets and then perform validation according to another statistical framework suggested by Jaffe *et al* [19]. All the 20 candidates have strong statistical significance (with pvalue less than 0.001).

### FastDMA Finds Essentially the Same Differentially Methylated Probes as IMA does but Performs with much Higher Computational Efficiency

We compared FastDMA and IMA (under Student’s t-test mode) on the three cancer DNA methylation with single probe analysis. The thresholds for identifying differentially probes were set as pvalue <0.01 and FDR <0.05. Table 2 shows the numbers of identified probes by IMA and FastDMA under different contexts. It is shown that more than 98% identified probes are overlapped in FastDMA and IMA, which indicates the correctness.

**Figure 4:**
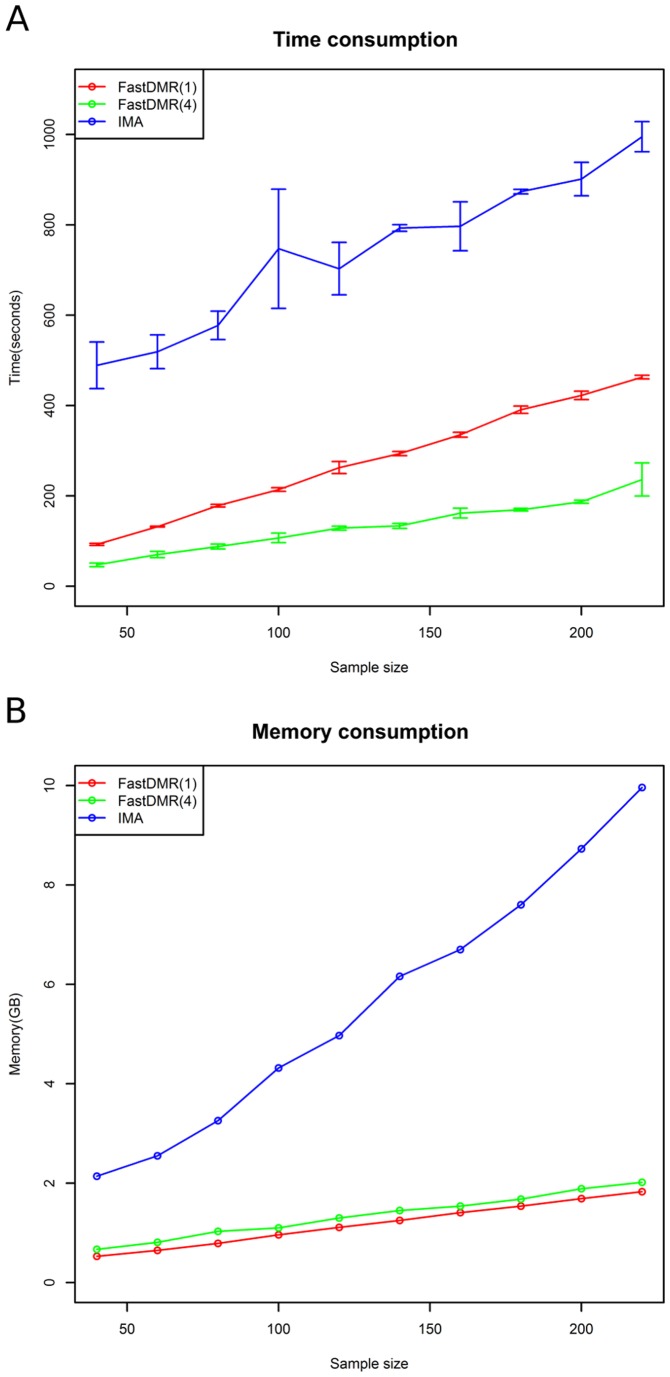
The comparison of computational efficiency between FastDMA and IMA. FastDMA was tested under both the single-thread and four-parallel-thread mode. **A)** The comparison on the average time consumption (calculated by repeatedly running the software three times). B) The comparison on the memory consumption.

To compare the computational efficiency, we ran them on a set of testing datasets (generated from real data of TCGA) with different data sizes varying from 40 to 220. FastDMA can work in parallel manner, we ran FastDMA twice using either single thread or four parallel threads. To measure the time consumption exactly, we repeatedly ran the software three times on every testing dataset and calculated the average time consumption with standard deviation. As indicated in figure 4, FastDMA works with much higher efficiency.

## Discussion

As a promising platform to detect genome-wide DNA methylation state, Infinium HumanMethylation450 Beadchip begins to be widely used with relatively lower cost. FastDMA provides an integrative pipeline and a high computational efficiency solution for analyzing this kind of data. This software can benefit the community of DNA methylation, especially for the large-scale clinical study. FastDMA uses general input format. Except 450 K Beadchip, it can also be used to analyze the data generated from other platforms, such as the previous Infinium HumanMethylation27 Beadchip and the possibly future Infinium beadchips with the same technique.

It should be noted that FastDMA is not elaborately designed for the data normalization. Users can use any other sophisticated normalization procedure (such as in ref. [20]) rather than the simply quantile normalization if this step is crucial for the following data analysis. Serious concerns about DNA methylation array are raised that the platform may contain potential technical artifacts [21]. To solve this problem, we design a command line parameter to filter out probes with genomic variants or co-hybridizing according to a recent work [22]. Also, any biological interpretations for the differential methylated probes should be made carefully.

FastDMA can smoothly run under major Linux/Unix systems using command line. For easier visualization of the results, the software formats the outputs as widely-used BED files that can be directly loaded into any genome browser tools. In the future, we will develop a graphic user interface for boarder users with less computer background.
